# How to Improve the Drafting of Health Profiles

**DOI:** 10.3390/ijerph19063452

**Published:** 2022-03-15

**Authors:** Margherita Napolitani, Giovanni Guarducci, Gulnara Abinova, Gabriele Messina, Nicola Nante

**Affiliations:** 1 Post Graduate School of Public Health, University of Siena, 53100 Siena, Italy; napolitani2@student.unisi.it (M.N.); giovanni.guarducc@student.unisi.it (G.G.); gabriele.messina@unisi.it (G.M.); 2 San Michele Private Hospital, 17031 Albenga, Italy; abinovagula@libero.it; 3 Department of Molecular and Developmental Medicine, University of Siena, 53100 Siena, Italy

**Keywords:** comorbidity, CIRS, risk adjustment, health profiles

## Abstract

Delineating patients’ health profiles is essential to allow for a proper comparison between medical care and its results in patients with comorbidities. The aim of this work was to evaluate the concordance of health profiles outlined by ward doctors and by epidemiologists and the effectiveness of training interventions in improving the concordance. Between 2018 and 2021, we analyzed the concordance between the health profiles outlined by ward doctors in a private hospital and those outlined by epidemiologists on the same patients’ medical records. The checks were repeated after training interventions. The agreement test (Cohen’s kappa) was used for comparisons through STATA. The initial concordance was poor for most categories. After our project, the concordance improved for all categories of CIRS. Subsequently, we noted a decline in concordance between ward doctors and epidemiologists for CIRS, so a new training intervention was needed to improve the CIRS profile again. Initially, we found a low concordance, which increased significantly after the training interventions, proving its effectiveness.

## 1. Introduction

The World Health Organization (WHO), in 1948, defined health as ‘a state of complete physical, mental and social well-being and not merely the absence of disease’. This definition focuses not only on merely biomedical aspects but also on the importance of mental and social well-being and implies the need to measure health, not only in terms of frequency of illness but also in terms of quality of life [1,2]. Nowadays, there is a need for a more stringent and up-to-date epidemiological definition that takes into account the special needs of an aging population. The concepts and relationships between health, well-being, and happiness need to be better specified. According to Alan J. Card, “good health and bad health are not a dichotomy, but a continuum. The absence of disease or disability is neither sufficient nor necessary to produce good health” [3]. In contemporary society, in which chronic and degenerative diseases have become increasingly important, this definition has practical implications in relation to the objectives of value-based care. In particular, it highlights the need for patient-centered care based on achievable goals, aiming at a better and lasting state of health, as opposed to the utopian goal of complete well-being required by the WHO definition. Obviously, the above cannot ignore the need to include measures of general health status [3]. As Lord W. Thomson of Kelvin said, “you can only say you know a phenomenon when you can measure it namely describe it in numbers” [4].

To measure the state of health, there are various instruments, generic or specific, objective or subjective [5]. Generic instruments assess health as a whole, while specific instruments focus on specific aspects (e.g., psychological health, level of pain, mobility, etc.) [6,7].

Health-related quality of life (HRQoL) can be calculated using perceived health measurement tools, such as the EuroQol-5D questionnaire (EQ 5D-5L) [8,9,10], short form-36 (SF-36) [11,12,13], Geriatric Scale Depression (GDS), etc. [14]. These instruments depend on the subjective perception of the patient, unlike those considered ‘objective’, which focus on externally measurable aspects and reproducible instrumental assessments. These include the Cumulative Illness Rating Scale (CIRS) [15] and Charlson comorbidity index (CCI) [16]: they are part of the isogravity patient classification systems used for outcome standardization. In Italy, their use is provided for by Ministerial Decree no. 70/2015, which, in outlining the general standards of quality of hospital care, establishes that there must be “documented and formalized presence of systems or activities of (...) evaluation and continuous improvement of clinical activities” through the “measurement of clinical performance and outcomes (...) and evaluation of the quality perceived by citizens/patients” [17].

Both of these tools outline a patient’s health profile by considering their comorbidities. The assessment of comorbidities is important because they may influence the treatments offered to patients, complications, survival and affect the use of resources [18,19]. 

It is therefore essential to know how to use these tools to make comparisons that are comparable, staging for equal levels of clinical severity [20]. In fact, failure to use them can lead to misinterpretation of clinical outcomes [21]. It is therefore essential that health care systems implement systematic policies to evaluate performance through the use of tools and methodologies that can make measurable and documentable the choices made by professionals in addressing care problems and the health outcomes obtained [22,23].

These ‘objective’ methods inevitably suffer from the subjectivity of the operator; so, the staff must be adequately trained and updated to make the results reproducible [24].

The aim of the work was:

1. To evaluate the concordance of health profiles outlined on the same case record with the CIRS tool, by ward doctors and a team of epidemiologists in charge of controls;
2. To evaluate the effectiveness of training interventions aimed at improving the drafting of the CIRS.

## 2. Materials and Methods

### 2.1. Study Setting and Participants

The study was carried out in a private clinic (100 beds), whose activity is mainly aimed at the hospitalization of patients for post-surgical orthopedic rehabilitation in agreement with the National Health Service (NHS). In addition, a part of the activity is dedicated to the admissions of internist-geriatric patients in a solvent regime. The CIRS health profile is compiled for each patient admitted to the clinic. The number of medical records in the early stages of the study was higher because the CIRS tool had recently been introduced and we were not sure that the compilation had been done for all patients. Once the project was fully operational, we did not need such a large number of medical records for our analysis, as all of them had the CIRS health profile.

### 2.2. Cumulative Illness Rating Scale (CIRS)

The CIRS is a tool used to outline a patient’s health profile by measuring the medical condition in 14 organs or systems (categories). Each category is given a severity of impairment score, ranging from one (none) to five (maximum). For patients with more than one disease in any one organ system, only the most severe disease is rated. Two indices are produced from the scores of categories:

- Severity index (SI): it represents the arithmetic mean of the scores of the first thirteen categories;
- Comorbidity index (CI): it represents the total number of categories in which the score is equal to or greater than three, excluding the 14th category (psychiatric disorders) [25,26].

### 2.3. Study Design and Project

**Figure 1:**
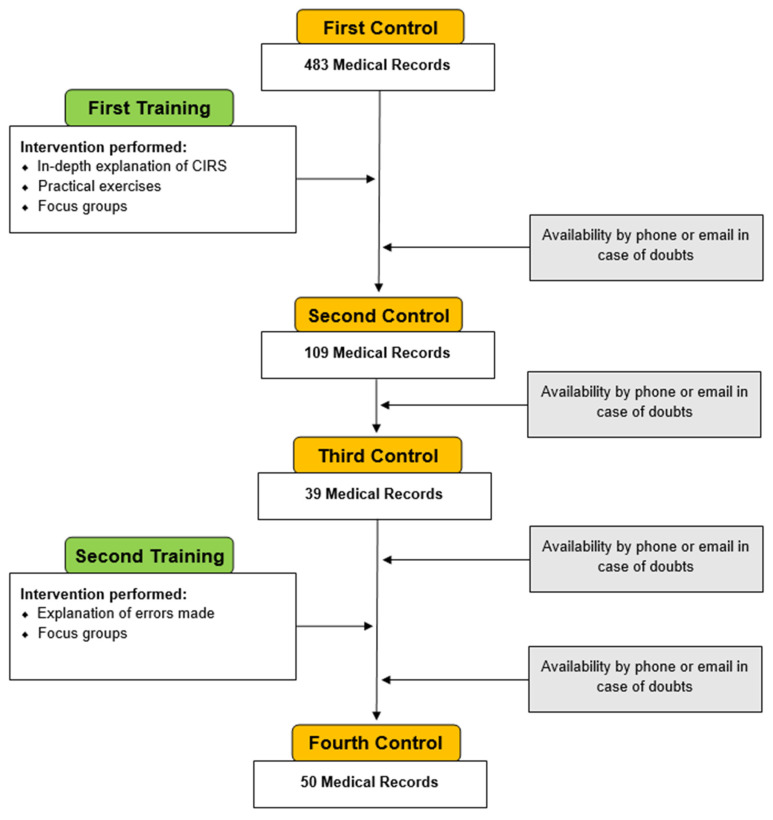
Flow chart of our project.

Figure 1 shows the flow chart of our project. It is divided into four phases of the CIRS control and two training phases. The training phases were developed by our Laboratory for Planning and Organization of Health Services, taking cues from some works in which healthcare professionals were trained on completing the CIRS [24,25].

In the first phase of control, in the period between January and November 2018, we conducted a retrospective study of 483 medical records of patients admitted between January and December 2017 in a private clinic. These were rehabilitation and geriatric case histories. For each patient, our epidemiological team, using the CIRS, traced the health profile. These profiles were compared with the CIRS outlined in real-time during patient admissions by the physicians of the clinic.

At the end of November 2018, after the first control started, the first training began. As part of a seminar in the clinic, we explained the CIRS guidelines to the doctors, and we provided a summary outline to facilitate memorization. Practical exercises on medical records were carried out, and we encouraged said staff to express their doubts and difficulties. Finally, a focus group was held to discuss the results of the practice exercise and to clarify any questions. In the follow-up period, training continued via email and telephone contact.

In the second control, from April to June 2019, in order to assess the effectiveness of the training intervention, we evaluated the concordance between the CIRS profiles we produced and those produced by the physicians of the private clinic. A sample of 109 new medical records of patients admitted to the private clinic from December 2018 to February 2019 was obtained.

During the third control, in May 2021, in order to assess the stability of the improvements achieved over time, we analyzed the concordance of the CIRS health profiles derived from 39 inpatient records between September and December 2020. 

After this control, we organized a second training based on distance learning interventions, focusing on the main critical issues identified. Another focus group was held to clarify any concerns that came up from the learning interventions.

Finally, in the fourth control, in November 2021, we analyzed the concordance between our CIRS and those of the doctors at the clinic, on a sample of 50 patient records, admitted in October 2021.

**Figure 2:**
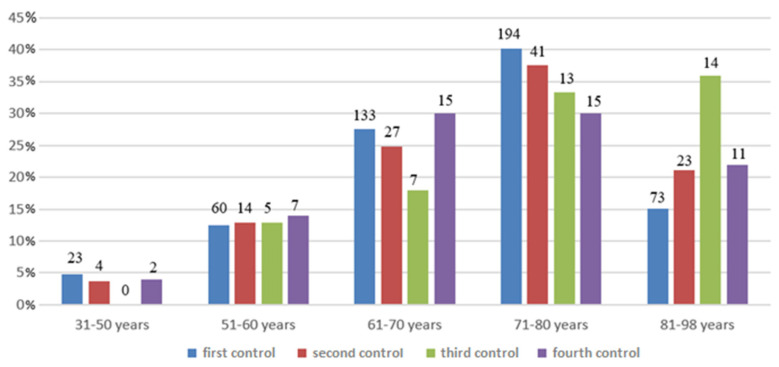
Sample divided for control and age.

Throughout the project, but especially between the first training and second control and between the third and fourth control, we kept in touch with the doctors in the clinic, by email or telephone. We had clarifying interpretative doubts, but also suggestions on how to compile the medical record in order to create automatisms that would make the tracking of the CIRS’s health profiles increasingly uniform (Figure 2).

### 2.4. Data Analysis

For the comparisons of the health profiles delineated by the physician of the clinic and by the epidemiological team, we used Cohen’s kappa (κ), which measures the inter-rater agreement between categorical scales when there are two raters. To interpret the results of Cohen’s kappa, we referred to the following intervals: from 0.01 to 0.20 slight agreement, from 0.21 to 0.40 fair agreement, from 0.41 to 0.60 moderate agreement, from 0.61 to 0.80 substantial agreement and from 0.81 to 1.00 almost perfect or perfect agreement [27,28]. Finally, the ANOVA test was used to test for differences between the means of three or more groups, and the Student’s *t*-test was used to assess differences between two controls. We used STATA software SE/14.0 (StataCorp LLC, College Station, TX, USA) to calculate them. This identified the main differences and systematic errors made when using the scale. Moreover, our results are reported graphically on a model that we have elaborated ad hoc in order to make the reading of the health profiles obtainable from the CIRS cards immediate.

## 3. Results

In the first control of our project, we analyzed 483 medical records. The mean age was 70.5 ± 10.5 years (minimum age 31 years, maximum age 93 years), Figure 1. The mean hospital stay was 16 days (a minimum of 1 day, maximum 143 days). Only for 314 medical records, it was possible to find the CIRS values calculated by the clinic physicians. 

**Figure 3:**
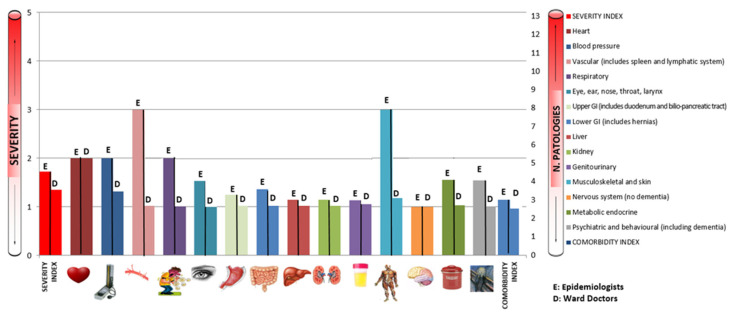
Concordance between epidemiologists and ward doctors (first control).

Figure 3 and Table 1 shows levels of agreement between CIRS values reported by the pool of epidemiologists and ward doctors before the training.

When analyzing the concordance between health profiles outlined by the epidemiological team and those outlined by the ward doctors, we found a moderate concordance for the category of heart disease (k = 0.49; *p* < 0.01). A slight agreement was found on respiratory diseases (k = 0.12; *p* < 0.01) and for the category of central nervous system diseases (k = 0.17; *p* < 0.01). A fair concordance was found for eight categories: blood pressure (k = 0.25; *p* < 0.01), sense organs (k = 0.23; *p* < 0.01), pathologies of the upper gastro-intestinal (GI) tract (k = 0.40; *p* < 0.01) and lower GI tract (k = 0.34; *p* < 0.01), kidney disease (k = 0.31; *p* < 0.01), genitourinary pathologies (k = 0.33; *p* < 0.01), the endocrine system and breast (k = 0.33; *p* < 0.01) and psychiatric disease (k = 0.30; *p* < 0.01). No agreement was found between our assessments and the health profile outlined by the clinic doctors for the categories of vascular system, liver system and musculoskeletal and skin (k < 0.01). Concerning the synthetic indicators, slight concordance was found for the total CIRS score (k = 0.03; *p* = 0.04), whereas no concordance was found for SI and CI (k < 0.01).

The first training described was conducted by three epidemiological doctors from our Laboratory for Planning and Organization of Health Services, involved five ward doctors, seven nurses, the physiotherapy coordinators as well as the health management of the structure. 

In the second control, we analyzed 109 medical records of patients admitted to the clinic between December 2018 and February 2019, at the time immediately following the training intervention. The mean age was 71.6 ± 11.09 (minimum age 38 years, maximum age 96 years), Figure 1. The mean hospital stay was 11.0 (a minimum of 1 day, maximum 36 days). Only for 14 medical records, the CIRS values calculated by the clinic doctors were not found.

**Figure 4:**
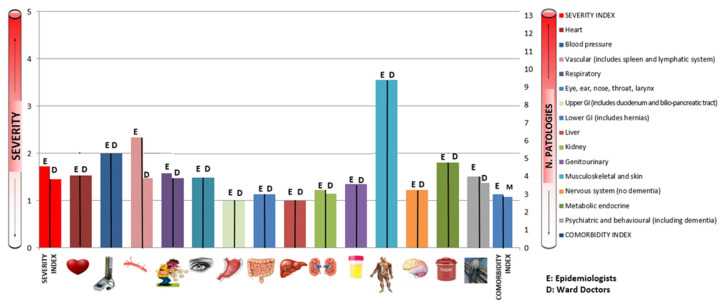
Concordance between epidemiologists and ward doctors (second control).

Figure 4 and Table 2 shows levels of agreement between CIRS values reported by the pool of epidemiologists and ward doctors after the seminar.

The concordance between, calculated from 95 records, was perfect or almost perfect for the categories of genitourinary diseases (k = 0.86; *p* < 0.01) and cardiac (k = 0.81; *p* < 0.01). The concordance was excellent for six categories: blood pressure (k = 0.74; *p* < 0.01), upper GI tract (k = 0.65; *p* < 0.01) and lower (k = 0.65; *p* < 0.01), the skeletal muscle and skin system (k = 0.68; *p* < 0.01), the endocrine system and breast (k = 0.67; *p* < 0.01) and the central nervous system (k = 0.71; *p* < 0.01). Concordance was moderate for the respiratory category (k = 0.48; *p* < 0.01), liver disease (k = 0.47; *p* < 0.01), kidney disease (k = 0.49; *p* < 0.01), the sense organs (k = 0.59; *p* < 0.01) and psychiatric disease (k = 0.50; *p* < 0.01). Concordance was fair for the vascular system (k = 0.24; *p* < 0.01). 

With regard to the synthetic indicators, a fair concordance was found for the CI (k = 0.30; *p* < 0.01), whereas for the CIRS total score (k = 0.15; *p* < 0.01) and for the SI (k = 0.18; *p* < 0.01), the concordance found was slight. As mentioned, additional training was therefore provided by telephone and email.

In May 2021 (third control), we analyzed a sample of 39 medical records from around the period between September and December 2020. The mean age was 74.4 ± 10.8 years (minimum age 58 years, maximum age 98 years), Figure 1. The mean hospital stay was 12.9 (minimum 3 days, maximum 26 days). For all medical records, the CIRS was calculated by the clinic doctor.

**Figure 5:**
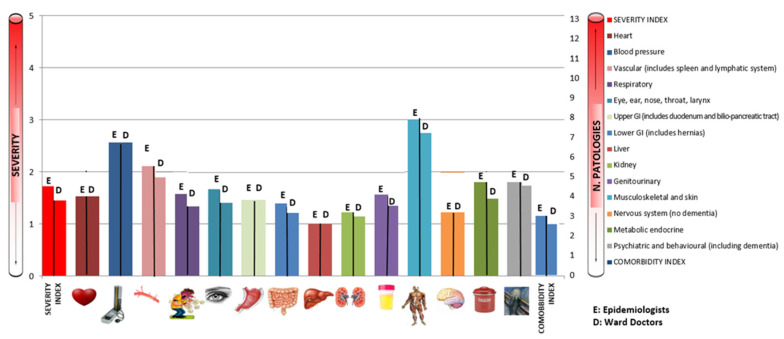
Concordance between epidemiologists and ward doctors (third control).

Figure 5 and Table 3 shows levels of agreement between CIRS values reported by the pool of epidemiologists and ward doctors.

The concordance between our assessments and those of the clinic doctors was calculated on 32 medical records, since in 7 cases the CIRS profiles calculated by the clinic physicians could not be found. We found substantial agreement for the category of heart disease (k = 0.64; *p* < 0.01), for the category assessing blood pressure (k = 0.63; *p* < 0.01) and for the category assessing central nervous system disease (k = 0.64; *p* < 0.01). Moderate concordance was found for five categories: the category of diseases of the upper GI tract (k = 0.41; *p* < 0.01), for the category of diseases of the liver (k = 0.43; *p* < 0.01) and kidney (k = 0.52; *p* < 0.01), for the endocrine system and breast (k = 0.43; *p* < 0.01) and for psychiatric (k = 0.44; *p* < 0.01). Fair agreement was found for the category of diseases of the vascular system (k = 0.33; *p* < 0.01), respiratory system (k = 0.21; *p* = 0.03), lower GI system (k = 0.35; *p* < 0.01), genitourinary diseases (k = 0.32; *p* < 0.01) and diseases of the sense organs (k = 0.22; *p* = 0.03). Slight agreement was found on the musculoskeletal-skin category (k = 0.03; *p* = 0.04). The concordance of the musculoskeletal-skeletal category deteriorated the fastest. Concerning the synthetic indicators, the concordance found was slight: total CIRS (k = 0.16; *p* < 0.01), SI (k = 0.13; *p* < 0.01) and CI (k = 0.06; *p* = 0.2).

Finally, in November 2021 (fourth control), we analyzed a sample of 50 medical records of patients admitted to the clinic during October 2021. The mean age was 70.9 ± 11.2 years (minimum age 34 years, maximum age 90 years), Figure 1. The mean hospital stay was 12.5 days (the minimum hospital stay was 8 days, and the maximum hospital stay was 15 days). CIRS values were calculated by the clinic doctors and could be found for all medical records. 

**Figure 6:**
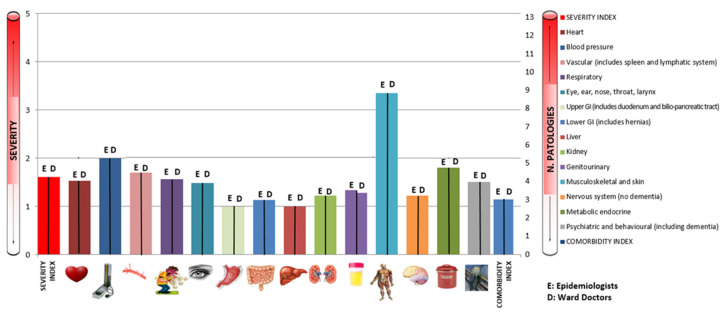
Concordance between epidemiologists and ward doctors (fourth control).

Figure 6 and Table 4 shows levels of agreement between CIRS values reported by the pool of epidemiologists and ward doctors.

The concordance was perfect or almost perfect for the categories of the upper GI system (k = 1; *p* < 0.01), the renal system (k = 1; *p* < 0.01), for heart disease (k = 0.94; *p* < 0.01), for assessing blood pressure (k = 0.97; *p* < 0.01), for the respiratory system (k = 0.82; *p* < 0.01) and for liver diseases (k = 0.88; *p* < 0.01). In particular, for the upper GI and kidney categories, the epidemiological team and the ward doctors assigned exactly the same values. Concordance was substantial for the categories of diseases of the vascular system (k = 0.74; *p* < 0.01), sense organs (k = 0.77; *p* < 0.01), lower GI tract (k = 0.67; *p* < 0. 01), musculoskeletal-skeletal system (k = 0.78; *p* < 0.01), central nervous system (k = 0.67; *p* < 0.01), endocrine (k = 0.75; *p* < 0.01) and psychiatric (k = 0.74; *p* < 0.01). Moderate concordance was found for the category assessing genitourinary disorders (k = 0.59; *p* < 0.01).

According to the synthetic indicators, the concordance found was almost perfect for the CI (k = 0.83; *p* < 0.01), while it was substantial for the calculation of the total CIRS score (k = 0.70; *p* < 0.01) and for the SI (k = 0.73; *p* < 0.01). 

Table 5 shows the levels of agreement between all phases of control. The ANOVA test shows a significant difference between all groups (*p* = 0.00). Specifically, the values of the second control, after the training intervention, increased compared to the values of the first control (*p* < 0.005). The same occurred between the third and fourth controls (*p* = 0.00). A decrease in values occurred between the second and third controls (*p* < 0.05).

## 4. Discussion

The functions of the CIRS are to make an outline of a patient’s health profile possible and to assess deviations over time or following therapeutic interventions (outcome measures), and allows for a comparison, by standardization, of the results of medical treatment in patients with different pathologies, staging them according to levels of severity (risk adjustment).

The instrument has been widely used in different settings, demonstrating extreme versatility: geriatric, psychiatric, oncological, in the context of general practice [24,29,30], etc. In our case, we used it for rehabilitation and geriatric admissions.

Fortin et al. [25] in a general practice setting, have shown that the presence of comorbidities has negative effects on the perception of quality of life. The relationship with the health-related quality of life of our case series, although studied, was not among the objectives of this report. Hall et al. [18] studied a sample of 379 patients with squamous cell carcinoma of the head and neck and showed that the CIRS scale is able to stratify patients by survival. Corbi et al. [31], assessing a sample of 200 Italian over-65s who had applied for an accompanying pension, showed that the CIRS scale can be useful in saving financial resources by reducing the risk of incorrectly granting the allowance, as it can improve the accuracy of impairment assessment in the social security system. The CIRS is considered an “objective” tool, but it inevitably suffers, in our view, from the subjectivity and training of the compiling staff. Fortin et al. [25] conducted a study in which two nurses interviewed 48 adult patients recruited in a primary care setting and statistically significant differences were found between the mean CIRS scores of the two evaluators (11.5 ± 4.7 vs. 10.1 ± 4.2; *p* < 0.01). In the first control of our project, we also highlighted that the subjectivity of the operator can significantly influence the results of this instrument. In fact, the doctors in the private clinic, despite having professional experience and being in direct contact with the patients, were not able to outline the health profiles of their patients in a way that could be reproduced by other operators. In fact, from the medical records themselves, the epidemiological team expert in CIRS outlined health profiles that were not in acceptable agreement with those calculated by the ward doctors, with the exception of the cardiac category. The moderate concordance found for this category can at least partly be explained by the greater attention that ward doctors pay to cardiovascular diseases. In fact, in the internist wards, there has always been particular attention to this type of pathology, which is one of the most frequent in geriatric patients. It is useful to reiterate how important it is, in order to increase the concordance of the evaluations, to teach practitioners to pay detailed attention to each category to be examined. The training on the CIRS instrument helps clinicians to assess their patients globally, without focusing exclusively on the acute problem that is the main reason for admission. In fact, when clinical conditions deteriorate, it is almost never due to a single problem [32]. A comprehensive approach to the patient, focusing on all organs and systems, provides a more complete picture and allows for more targeted and effective care [33]. For this purpose, the medical record must be compiled in detail and the staff analyzing the records must be properly trained, as also emphasized by Hudon et al. [24], who demonstrated that reliable CIRS profiles can be delineated by experienced nurses by consulting medical records.

Not all assessment instruments require staff to be particularly well trained. Ariza-Vega et al. [34], in calculating the reliability of the assessments of two physiotherapists, who had independently used the Spanish version of the cumulated ambulation score (CAS) in 60 patients with a hip fracture within the first postoperative week, showed overlapping results. The CAS score is probably easier to apply because it consists of three tasks with scores ranging from 0 to 2 points (instead of the five scores of the CIRS), but it captures fewer nuances. The effectiveness evaluation conducted after our training intervention (first training) showed a marked increase in the reliability of the CIRS. This probably occurred not only because the guidelines for filling in the forms were explained, but also because staff motivation increased, and they became more aware of the usefulness of the tool. If healthcare professionals are not motivated, they may perceive it as an additional burden on their already busy work schedule and fill it out in a superficial and hasty manner. In fact, about half of physicians see their own positive attitude and motivation for change as facilitating it [35].

The concordance of the findings concerning respiratory diseases increased the least after the seminar. This is probably because there is little attention and little habitual recording of smoking data in the file. Too often, doctors forget to investigate patients’ habits and lifestyles. This is in spite of scientific evidence showing that smoking causes a wide variety of diseases [36].

The check carried out on the CIRS produced by the ward doctors between September and December (2020) revealed a deterioration in the concordance of almost all categories. This decrease in agreement was higher for the musculoskeletal-cutaneous, respiratory, and vascular categories.

When an operator is aware that they are being watched, he is more committed and gives the best of his abilities: the so-called ‘Hawthorne effect’ [37]. Without control, improvements do not last, as continuous monitoring and communication help achieve the desired goal [38]. Healthcare organizations need to be aware of these mechanisms and to design systems of control and continuous training for their employees. We promptly organized remote meetings, telephone consultations and again provided material via email, and also trained and involved the newly recruited staff, as it is necessary to train them thoroughly as soon as they start their employment [39]. We have focused on the difficulties identified in the analysis and have tried to renew the enthusiasm and participation of staff. We asked everyone for their input, encouraging them to report any problems or difficulties and to suggest improvements: having feedback is an important component of most quality improvement interventions [40]. We responded to the difficulties expressed by the employees, and, for this reason, we increased the efficiency of the computer workstations and the functionality of the software. In fact, the lack of advanced computing resources can be a barrier to achieving the objective [36]. In order to obtain reliable and stable results over time, we carried out the check after a few months, to allow the production of settled data that would be indicative of the ability of the clinic’s staff to outline the health profiles of the patients admitted. Certainly, in order to maintain a high level of agreement, it will be necessary to carry out the training interventions at a fixed frequency because this allows to achieve greater effectiveness [41]. Although our goal was very ambitious, we proved that proper training, great determination and active involvement of staff is effective in achieving a substantial or near-perfect match in most categories. In fact, in a team environment, having a clear, specific, and accepted goal directs the action of the various team members and motivates the strategy development to reach it [42,43]. Future research will be needed to confirm the practical usefulness of our proposed training interventions or whether implementation strategies will be needed, should new barriers arise.

### Limitations

This work has some limitations. The staff of the private clinic is made up in part by experienced doctors, but in part by new graduates with their first work experience, with frequent turnover; this partially invalidates the stability of the results acquired with the training interventions. The distance between the clinic and our Health Service Research Laboratory (whose epidemiological team carried out the training and quality control activities for the compilation of the CIRS forms/profiles) may be perceived as “distant” attention; it certainly does not facilitate the transmission of skills to newcomers. In the early months of 2020, as is well known, the COVID-19 epidemic affected the entire hospital organization in northern Italy. From April to June, the clinic that was under study suspended its usual orthopedic rehabilitation admissions (under the National Health Service) and internist and geriatric admissions (in a solvent setting) to devote itself exclusively to COVID-19 patients; since July, normal activity has gradually restarted. As mentioned, in the first pandemic period (April–June 2020), the entire clinic’s activity was only for COVID-19 patients, for whom, due to the emergency, CIRS profiles were not calculated. As soon as the clinic’s normal activity resumed, the routine compilation of CIRS also resumed, but the project restarted without due attention.

## 5. Conclusions

The CIRS is useful for outlining the health profiles of patients and populations and as a risk adjustment tool. In order to be reliably used in these functions, adequate training of the personnel involved is required, and constant quality control of its compilation is necessary. At the beginning of our project, the concordance between the quality of compilation of the CIRS forms expressed by our team and that of the ward doctors appeared poor or weak for most categories: this, in itself, calls into question the presumed “objectivity” of the tool. After appropriate training, the concordance between the surveyors increased considerably. In the absence of updating and monitoring, the concordance tends to decrease, but can quickly return to reliable levels after further targeted training.

## Figures and Tables

**Table 1:** Levels of agreement between the CIRS values reported by the pool of epidemiologists and ward doctors (first control).

CATEGORY	Agreement	Kappa	95% C.I.	*p* Value
HEART	86%	0.49	0.4116–0.5684	<0.01
BLOOD PRESSURE	42.4%	0.25	0.2108–0.2892	<0.01
VASCULAR	8.3%	−0.001	−0.0206–0.0186	0.55
RESPIRATORY	75.5%	0.12	0.0612–0.1788	<0.01
SENSE ORGANS	66.2%	0.23	0.1516–0.3084	<0.01
UPPER G.I.	93.0%	0.40	0.3216–0.4784	<0.01
LOWER G.I.	93.3%	0.34	0.2616–0.4184	<0.01
HEPATIC-PANCREATIC	87.9%	−0.03	−0.1084–0.0484	0.8
RENAL	94.9%	0.31	0.212–0.408	<0.01
GENITOURINARY	74.5%	0.33	0.2516–0.4084	<0.01
MUSCULOSKELETAL	20.38%	−0.01	−0.0296–0.0096	0.6
NEUROLOGICAL	94.6%	0.17	0.0916–0.2484	<0.01
ENDOCRINE	66.6%	0.33	0.2516–0.4084	<0.01
PSYCHIATRIC	71.7%	0.30	0.2412–0.3588	<0.01
TOTAL CIRS	9.5%	0.03	0.0104–0.0496	0.04
SEVERITY INDEX	0.3%	0.00	0	0.3
COMORBIDITY INDEX	14.7%	0.00	−0.0392–0.0392	0.2

**Table 2:** Levels of agreement between the CIRS values reported by the pool of epidemiologists and ward doctors (second control).

CATEGORY	Agreement	Kappa	95% C.I.	*p* Value
HEART	93.7%	0.81	0.6728–0.9472	<0.01
BLOOD PRESSURE	84.2%	0.74	0.5832–0.8968	<0.01
VASCULAR	44.2%	0.24	0.1224–0.3576	<0.01
RESPIRATORY	80.0%	0.48	0.3428–0.6172	<0.01
SENSE ORGANS	76,8%	0.59	0.4332–0.7468	<0.01
UPPER G.I.	94.7%	0.65	0.4932–0.8068	<0.01
LOWER G.I.	93.7%	0.65	0.5128–0.7872	<0.01
HEPATIC-PANCREATIC	91.6%	0.47	0.3132–0.6268	<0.01
RENAL	96.8%	0.49	0.3332–0.6468	<0.01
GENITOURINARY	95.7%	0.86	0.7228–0.9972	<0.01
MUSCULOSKELETAL	82.1%	0.68	0.5232–0.8368	<0.01
NEUROLOGICAL	94.7%	0.71	0.5924–0.8276	<0.01
ENDOCRINE	80.0%	0.67	0.5328–0.8072	<0.01
PSYCHIATRIC	89.5%	0.50	0.3432–0.6568	<0.01
TOTAL CIRS	21.1%	0.15	0.0912–0.2088	<0.01
SEVERITY INDEX	24.8%	0.18	0.1212–0.2388	<0.01
COMORBIDITY INDEX	43.1%	0.30	0.2216–0.3784	<0.01

**Table 3:** Levels of agreement between the CIRS values reported by the pool of epidemiologists and ward doctors (third control).

CATEGORY	Agreement	Kappa	95% C.I.	*p* Value
HEART	87.5%	0.64	0.4048–0.8752	<0.01
BLOOD PRESSURE	75.0%	0.63	0.3948–0.8652	<0.01
VASCULAR	56.3%	0.33	0.1732–0.4868	<0.01
RESPIRATORY	71.9%	0.21	−0.0056–0.4256	0.03
SENSE ORGANS	56.3%	0.22	−0.0152–0.4552	0.03
UPPER G.I.	71.9%	0.41	0.1356–0.6844	<0.01
LOWER G.I.	71.9%	0.35	0.1932–0.5068	<0.01
HEPATIC-PANCREATIC	84.4%	0.43	0.1360–0.7240	<0.01
RENAL	87.5%	0.52	0.3044–0.7356	<0.01
GENITOURINARY	62.5%	0.32	0.1044–0.5356	<0.01
MUSCULOSKELETAL	34.4%	0.03	−0.1660–0.2260	0.04
NEUROLOGICAL	93.8%	0.64	0.4048–0.8752	<0.01
ENDOCRINE	65.7%	0.43	0.2340–0.6260	<0.01
PSYCHIATRIC	75.0%	0.44	0.2244–0.6556	<0.01
TOTAL CIRS	21.9%	0.16	0.0620–0.2580	<0.01
SEVERITY INDEX	18.8%	0.13	0.0516–0.2084	<0.01
COMORBIDITY INDEX	21.9%	0.06	−0.0772–0.1972	0.2

**Table 4:** Levels of agreement between the CIRS values reported by the pool of epidemiologists and ward doctors (fourth control).

CATEGORY	Agreement	Kappa	95% C.I	*p*
HEART	98.0%	0.94	0.7244–1.1556	<0.01
BLOOD PRESSURE	98.0%	0.97	0.7740–1.1660	<0.01
VASCULAR	94.0%	0.74	0.5048–0.9752	<0.01
RESPIRATORY	98.0%	0.82	0.5848–1.0552	<0.01
SENSE ORGANS	92.0%	0.77	0.4956–1.0444	<0.01
UPPER G.I.	100%	1.0	0.7844–1.2156	<0.01
LOWER G.I.	98.0%	0.67	0.4740–0.8660	<0.01
HEPATIC-PANCREATIC	98.0%	0.88	0.6056–1.1544	<0.01
RENAL	100%	1.0	0.7256–1.2744	<0.01
GENITOURINARY	86.0%	0.59	0.3940–0.7860	<0.01
MUSCULOSKELETAL	88.0%	0.78	0.5252–1.0348	<0.01
NEUROLOGICAL	98.0%	0.67	0.4740–0.8660	<0.01
ENDOCRINE	88.0%	0.75	0.5344–0.9656	<0.01
PSYCHIATRIC	96.0%	0.74	0.5440–0.9360	<0.01
TOTAL CIRS	80.4%	0.70	0.6020–0.27980	<0.01
SEVERITY INDEX	82.1%	0.73	0.6320–0.8280	<0.01
COMORBIDITY INDEX	89.3%	0.83	0.7124–0.9476	<0.01

**Table 5:** Levels of agreement for all phases of control.

CATEGORY	First Control	Second Control	Third Control	Fourth Control
HEART	86%	93.7%	87.5%	98.0%
BLOOD PRESSURE	42.4%	84.2%	75.0%	98.0%
VASCULAR	8.3%	44.2%	56.3%	94.0%
RESPIRATORY	75.5%	80.0%	71.9%	98.0%
SENSE ORGANS	66.2%	76.8%	56.3%	92.0%
UPPER G.I.	93.0%	94.7%	71.9%	100%
LOWER G.I.	93.3%	93.7%	71.9%	98.0%
HEPATIC-PANCREATIC	87.9%	91.6%	84.4%	98.0%
RENAL	94.9%	96.8%	87.5%	100%
GENITOURINARY	74.5%	95.7%	62.5%	86.0%
MUSCULOSKELETAL	20.38%	82.1%	34.4%	88.0%
NEUROLOGICAL	94.6%	94.7%	93.8%	98.0%
ENDOCRINE	66.6%	80.0%	65.7%	88.0%
PSYCHIATRIC	71.7%	89.5%	75.0%	96.0%
TOTAL CIRS	9.5%	21.1%	21.9%	80.4%
SEVERITY INDEX	0.3%	24.8%	18.8%	82.1%
COMORBIDITY INDEX	14.7%	43.1%	21.9%	89.3%
MEAN	58.79 ± 33.84	75.69 ± 24.78	62.16 ± 23.46	93.16 ± 6.18
